# Is cost effectiveness sustained after weekend inpatient rehabilitation? 12 month follow up from a randomized controlled trial

**DOI:** 10.1186/s12913-015-0822-3

**Published:** 2015-04-18

**Authors:** Natasha Kareem Brusco, Jennifer J Watts, Nora Shields, Nicholas F Taylor

**Affiliations:** School of Allied Health, Department of Rehabilitation, Sport and Nutrition, College of Science, Health and Engineering, La Trobe University, Bundoora, VIC 3086 Australia; Physiotherapy Services, Cabrini Health, 183 Wattletree Road, Malvern, VIC 3144 Australia; Deakin Health Economics, Population Health SRC, Faculty of Health, Deakin University, 221 Burwood Highway, Burwood, VIC 3125 Australia; Department of Allied Health, Northern Health, 1231 Plenty Road, Bundoora, VIC 3083 Australia; Allied Health Clinical Research Office, Eastern Health, Level 2, 5 Arnold Street, Box Hill, VIC 3128 Australia

**Keywords:** Rehabilitation, Economic evaluation, Randomized controlled trial, Allied health

## Abstract

**Background:**

Our previous work showed that providing additional rehabilitation on a Saturday was cost effective in the short term from the perspective of the health service provider. This study aimed to evaluate if providing additional rehabilitation on a Saturday was cost effective at 12 months, from a health system perspective inclusive of private costs.

**Methods:**

Cost effectiveness analyses alongside a single-blinded randomized controlled trial with 12 months follow up inclusive of informal care. Participants were adults admitted to two publicly funded inpatient rehabilitation facilities. The control group received usual care rehabilitation services from Monday to Friday and the intervention group received usual care plus additional Saturday rehabilitation. Incremental cost effectiveness ratios were reported as cost per quality adjusted life year (QALY) gained and for a minimal clinical important difference (MCID) in functional independence.

**Results:**

A total of 996 patients [mean age 74 years (SD 13)] were randomly assigned to the intervention (n = 496) or control group (n = 500). The intervention was associated with improvements in QALY and MCID in function, as well as a non-significant reduction in cost from admission to 12 months (mean difference (MD) AUD$6,325; 95% CI −4,081 to 16,730; *t* test *p* = 0.23 and *MWU p* = 0.06), and a significant reduction in cost from admission to 6 months (MD AUD$6,445; 95% CI 3,368 to 9,522; *t test p* = 0.04 and *MWU p* = 0.01). There is a high degree of certainty that providing additional rehabilitation services on Saturday is cost effective. Sensitivity analyses varying the cost of informal carers and self-reported health service utilization, favored the intervention.

**Conclusions:**

From a health system perspective inclusive of private costs the provision of additional Saturday rehabilitation for inpatients is likely to have sustained cost savings per QALY gained and for a MCID in functional independence, for the inpatient stay and 12 months following discharge, without a cost shift into the community.

**Trial registration:**

Australian and New Zealand Clinical Trials Registry November 2009 ACTRN12609000973213.

**Electronic supplementary material:**

The online version of this article (doi:10.1186/s12913-015-0822-3) contains supplementary material, which is available to authorized users.

## Background

The role of rehabilitation within a health service is to achieve and maintain optimal functioning for the patient [1]. Rehabilitation services are available across a variety of settings including acute hospitals, inpatient rehabilitation facilities or in the community [1,2]. There is emerging evidence of short term clinical and economic gains for the provision of additional weekend rehabilitation services for inpatients including likely cost savings associated with a reduction in hospital length of stay, as well as higher functional status and improved quality of life on discharge from rehabilitation [3,4].

There is an important distinction between achieving short term gains following an intervention and sustaining these gains over the medium and longer term [5]. The success of rehabilitation may initially focus on the point of discharge from the rehabilitation program, but there is also value in evaluating the sustainability of this success. Measuring success for inpatient rehabilitation may include reporting gains in functional status or quality of life using specific outcome measures [4], or it may take a broader view that reports the ability to return to the community, participate in the workforce and resume usual societal roles [6]. No matter the viewpoint, sustainability of the intervention is an important consideration to measuring the success of inpatient rehabilitation.

Another consideration in measuring the success and sustainability of rehabilitation is understanding the resource impact on the health system and the broader community beyond discharge from the rehabilitation facility. In the 12 months following discharge from rehabilitation, patients have a high rate of health care and community resource use, such as readmission to inpatient health services, visits to allied health professionals, and the use of informal care which can increase the burden on the community [7,8]. An economic evaluation limited to the perspective of the inpatient rehabilitation facility will miss the ongoing health and community resources utilized once the patient has been discharged to the community. Taking a broader health system perspective inclusive of private costs and a longer follow up period will capture these additional resources.

Providing additional Saturday rehabilitation is likely to be cost saving per quality adjusted life year (QALY) gained and for a minimal clinical important difference (MCID) in functional independence from the perspective of the individual health service including the 30 day period following discharge [3]. However, the resource impact on the broader health system is not known in the 12 month period following discharge and whether the cost and clinical outcome results favoring additional Saturday rehabilitation are sustained in the medium and longer term. The aim of this study was to determine from a health system perspective inclusive of private costs if the likely short-term cost effectiveness of providing an additional Saturday rehabilitation service to inpatients in addition to Monday to Friday compared to Monday to Friday rehabilitation alone, is sustained 12 months following discharge from rehabilitation.

## Methods

### Research design and intervention

An economic evaluation alongside a randomized controlled trial from a health system perspective inclusive of private costs from admission until 12 months following discharge, was completed comparing usual care Monday to Friday rehabilitation to Monday to Saturday rehabilitation. Saturday rehabilitation consisted of a one hour scheduled Physiotherapy session and a one hour scheduled Occupational Therapy session. Full details of the protocol [9], the clinical outcomes [4] and an economic evaluation of the clinical trial with a 30 day follow up period from the perspective of the health service [3] have been published elsewhere. The trial was registered with the Australian and New Zealand Clinical Trials Registry (ACTRN12609000973213) prior to patient recruitment. This economic evaluation follows the recommendations of the Consolidated Health Economic Evaluation Reporting Standards (CHEERS) Checklist [10].

The economic evaluation has taken a health system perspective inclusive of private costs. Out-of-pocket costs for access to medical, non-medical services and pharmaceuticals have been included in this analysis. Informal care was also included on the assumption that its availability meant that health system costs were reduced. For example, without the availability of informal care at discharge, length of stay may have increased, or patients may have been discharged with additional formal community-based health services. We do not consider this economic evaluation to be from a broader societal perspective as productivity changes have not been incorporated.

### Participants, recruitment and setting

Recruitment occurred from July 2010 until June 2011 and participants were included if they were 18 years or older and required acute inpatient rehabilitation at one of the two facilities participating in the trial [9]. The economic analysis included the initial rehabilitation admission and had a primary endpoint 12 months following discharge from rehabilitation, with an interim endpoint 6 months following discharge from rehabilitation.

### Administrative data

Administrative data were obtained from the primary health service for the initial rehabilitation admission and for any admission in the period 12 months following discharge. The data were at the individual level and included the reason for admission, diagnosis related group (DRG), length of stay, as well as resource use and cost. In addition, data from Medicare for medical services and pharmaceuticals (Pharmaceutical Benefits Scheme (PBS)) were obtained for each consenting participant for the 12 months following discharge (Tables 1 and 2).

**Table 1:** **Cost variables, resources used and average cost per patient for the control group and the intervention group for the period between the initial inpatient admission and 12 months following discharge (AUD$2012/13)**

	**Mean resource utilization per patient for the period between the initial inpatient admission and 12 months following discharge (SD; range)** ^**a**^		**Mean cost per patient for the period between the initial inpatient admission and 12 months following discharge (SD; range)** ^**a**^ **$2012/13**		**Difference between groups for resource utilization**	**Difference between groups for costs**
	**Intervention**	**Control**	**Intervention**	**Control**	**Intervention minus control (95% CI)**	**Intervention minus control (95% CI)**
	**(n = 496)**	**(n = 500)**	**(n = 496)**	**(n = 500)**		
Initial inpatient rehabilitation admission (length of stay)	21.2	23.1	13,320	14,302	−1.9	−981
	(15.7; 4 to 144)	(20.2; 1 to 236)	(10,041; 2,652 to 93,095)	(12,168; 1,459 to 137,387)	(−4.1 to 0.4)	(−2,408 to 445)
Hospital readmissions in primary health service (length of stay)	10.3	15.2	10,283	13,122	−4.9*	−2,838
	(24.5; 0 to 156)	(37.4; 0 to 260)	(24,105; 0 to 195,939)	(30,255; 0 to 212,390)	(−9.0 to −0.9)	(−6,340 to 664)
Hospital readmissions in other health services (length of stay)	2.4	2.9	2364	2829	−0.6	−465
	(12.6; 0 to 63)	(7.9; 0 to 175)	(8013; 0 to 67,095)	(12,146; 0 to 169,419)	(−1.9 to 0.8)	(−1,790 to 859)
Total Medicare claims	50.1	52.7	3,029	3,108	−2.6	−79
	(45.8; 0 to 282)	(58.4; 0 to 735)	(3,704; 0 to 30,129)	(4,369; 0 to 53,232)	(−9.3 to 4.2)	(−598 to 439)
Benefit paid			2,567	2,684		−117
			(2,533; 0 to 17,643)	(3,037; 0 to 36,847)		(−469 to 235)
Out-of-pocket			462	424		38
			(1,409; 0 to 12,845)	(1,620; 0 to 18,647)		(−157 to 232)
Total non-Medicare health care visits	27.0	29.8	1,479	1,669	−2.7	−190
	(49.1; 0 to 375)	(58.8; 0 to 759)	(3,016; 0 to 29,534)	(4,174; 0 to 59,634)	(−9.6 to 4.1)	(−642 to 262)
Physiotherapists	13.8	14.1	670	685	−0.3	−15
	(26.4; 0 to 360)	(21.4; 0 to 224)	(1,281; 0 to 17,460)	(1,037; 0 to 10,864)	(−3.4 to 2.8)	(−164 to 134)
Occupational therapists	2.5	2.9	105	122	−0.4	−17
	(9.9; 0 to 94)	(10.2; 0 to 128)	(417; 0 to 3,975)	(433; 0 to 5,413)	(−1.7 to 0.9)	(−71 to 38)
Other allied health	4.6	4.7	217	223	−0.1	−6
	(15.0; 0 to 156)	(15.2; 0 to 169)	(701; 0 to 7,335)	(712; 0 to 7,946)	(−2.0 to 1.8)	(−96 to 84)
Community nurse	6.1	8.0	487	639	−1.9	−152
	(30.6; 0 to 364)	(48.6; 0 to 732)	(2,439; 0 to 29,007)	(3,869; 0 to 58,333)	(−4.6 to 0.7)	(−363 to 59)
Total pharmaceuticals (PBS) claims	55.9	61.1	2,368	2,558	−5.2	−190
	(42.5; 0 to 259)	(46.1; 0 to 279)	(2,749; 0 to 37,430)	(3,453; 0 to 48,717)	(−10.9 to 0.5)	(−568 to 189)
Benefit paid			2,074	2,256		−182
			(2,678; 0 to 37,183)	(3,388; 0 to 48,093)		(−564 to 200)
Out-of-pocket			294	302		−8
			(212; 0 to 1,411)	(196; 0 to 1,314)		(−34 to 18)
Over-the-counter medications	3.0	3.0	81	72	0.04	9
	(2.8; 0 to 16)	(2.9; 0 to 15)	(152; 0 to 2,084)	(105; 0 to 999)	(−0.3 to 0.4)	(−8 to 26)
Formal (paid) carers (hours)	22.3	21.6	764	740	0.7	24
	(58.5; 0 to 494)	(52.0; 0 to 408)	(2,002; 0 to 16,915)	(1,781; 0 to 13,970)	(−6.3 to 7.7)	(−217 to 265)
Informal (unpaid) carers (hours)	822.7	869.8	28,170	29,784	−47.1	−1,614
	(2,025; 0 to 9,906)	(52.0; 0 to 11,232)	(69,351; 0 to 339,181)	(69,902; 0 to 384,584)	(−180 to 86)	(−6,160 to 2,933)
Total			61,859	68,184		−6,325
			(79,798; 4,845 to 463,656)	(82,922; 4,845 to 463,656)		(−16,730 to 4,081)
						*t*-test p = 0.234
						MWU p = 0.056

Sources of price information: hospital admissions in primary health service: variable cost; hospital admissions in other health services; acute hospital $1,065/night, rehabilitation hospital $751/night; Medicare claims: variable; physiotherapy: $48.50/visit ^b^; occupational therapy: $42.29/visit ^b^speech therapy: $46.59/visit ^b^other allied health: $47.02/visit ^b^community nurse: $79.69/visit ^b^Pharmaceutical Benefits Scheme (PBS) claims: variable; over-the-counter medications: variable; formal and informal carers: $34.24/hour^b^.

*p ≤ 0.05. ^a^death weights have been applied to this data set to represent time in the study pertaining to patient death. ^b^TAC [13]. MWU, Mann–Whitney *U* test.

**Table 2:** **Cost variables, resources used and average cost per patient for the control group and the intervention group for the period between the initial inpatient admission and 6 months following discharge (AUD$2012/13)**

	**Mean resource utilization per patient for the period between the initial inpatient admission and 6 months following discharge (SD; range)** ^**a**^		**Mean cost per patient for the period between the initial inpatient admission and 6 months following discharge (SD; range)** ^**a**^ **$2012/13**		**Difference between groups for resource utilization**	**Difference between groups for costs**
	**Intervention**	**Control**	**Intervention**	**Control**	**Intervention minus control (95% CI)**	**Intervention minus control (95% CI)**
	**(n = 496)**	**(n = 500)**	**(n = 496)**	**(n = 500)**		
Initial inpatient rehabilitation admission (length of stay)	21.2	23.1	13,320	14,302	−1.9	−981
	(15.7; 4 to 144)	(20.2; 1 to 236)	(10,041; 2,652 to 93,095)	(12,168; 1,459 to 137,387)	(−4.1 to 0.4)	(−2,408 to 445)
Hospital readmissions in primary health service (length of stay)	5.8	9.5	5,550	8,019	−3.6*	−2,469*
	(17.1; 0 to 144)	(26.8; 0 to 195)	(15,084; 0 to 167,322)	(21,529; 0 to 176,202)	(−6.5 to −0.7)	(−4,849 to −1,214)
Hospital readmissions in other health services (length of stay)	1.3	1.8	1,360	1,717	−0.5	−357
	(5.7; 0 to 63)	(9.7; 0 to 160)	(5,912; 0 to 67,095)	(9,215; 0 to 157,840)	(−1.4 to 0.5)	(−1,319 to 606)
Total Medicare claims	17.5	16.7	1,059	975	0.8	84
	(24.7; 0 to 194)	(22.3; 0 to 191)	(2,102; 0 to 24,019)	(1,897; 0 to 26,724)	(−2.3 to 3.8)	(−173 to 340)
Benefit paid			881	835		46
			(1,393; 0 to 12,932)	(1,266; 0 to 11,689)		(−121 to 214)
Out-of-pocket			178	140		37
			(836; 0 to 11,087)	(823; 0 to 15,035)		(−69 to 144)
Total non-Medicare health care visits	17.2	18.4	934	1,022	−1.3	−89
	(32.5; 0 to 380)	(33.5; 0 to 369)	(1,911; 0 to 17,370)	(2,264; 0 to 29,304)	(−5.5 to 3.0)	(−357 to 180)
Physiotherapists	9.0	9.1	439	441	−0.1	−3
	(16.3; 0 to180)	(13.2; 0 to 108)	(788; 0 to 8,730)	(638; 0 to 5,238)	(−1.9 to 1.8)	(−94 to 89)
Occupational therapists	1.9	2.1	82	90	−0.2	−8
	(8.3; 0 to 140)	(7.3; 0 to 72)	(351; 0 to 5,921)	(308; 0 to 3,045)	(−1.2 to 0.8)	(−50 to34)
Other allied health	2.5	2.6	116	120	−0.1	−4
	(9.2; 0 to 100)	(9.4; 0 to 88)	(432; 0 to 4,659)	(442; 0 to 4,138)	(−1.3 to 1.1)	(−60 to 52)
Community nurse	3.7	4.7	297	371	−0.9	−74
	(18.3; 0 to 182)	(25.4; 0 to 366)	(1,462; 0 to 14,504)	(2,027; 0 to 29,167)	(−4.0 to 2.1)	(−268 to 120)
Total pharmaceuticals (PBS) claims	28.6	31.4	1,246	1,325	−2.8	−79
	(21.9; 0 to 259)	(24.1; 0 to 279)	(1,644; 0 to 37,430)	(1,848; 0 to 48,717)	(−5.8 to 0.2)	(−194 to 35)
Benefit paid			1,087	1,168		−81
			(1,608; 0 to 37,183)	(1,810; 0 to 48,093)		(−193 to 31)
Out-of-pocket			158	157		2
			(126; 0 to 1,411)	(114; 0 to 1,314)		(−14 to 17)
Over-the-counter medications	1.4	1.5	39	37	−0.01	2
	(1.5; 0 to 8)	(1.5; 0 to 8)	(80; 0 to 1,117)	(67; 0 to 931)	(−0.2 to 0.2)	(−7 to 12)
Formal (paid) carers (hours)	11.3	11.2	388	385	0.1	3
	(32.5; 0 to 247)	(27.1; 0 to 182)	(1,113; 0 to 8,457)	(929; 0 to 6,232)	(−3.7 to 3.9)	(−128 to 134)
Informal (unpaid) carers (hours)	387.7	462.4	13,274	15,833	−74.7*	−2,559*
	(1,055; 0 to 4,654)	(1,146; 0 to 5,616)	(36,135; 0 to 159,353)	(39,232; 0 to 192,292)	(−146.7 to −2.8)	(−5,022 to −96)
Total			37,170	43,615		−6,445
			(44,351; 3,713 to 231,733)	(49,806; 3,723 to 256,182)		(−9,522 to −3,368)
						*t*-test p = 0.036*
						MWU p = 0.010*

Sources of price information: hospital admissions in primary health service: variable cost; hospital admissions in other health services; acute hospital $1,065/night, rehabilitation hospital $751/night; Non-Medicare claims: variable; physiotherapy: $48.50/visit ^b^occupational therapy: $42.29/visit ^b^speech therapy: $46.59/visit ^b^other allied health: $47.02/visit ^b^community nurse: $79.69/visit ^b^Pharmaceutical Benefits Scheme (PBS) claims: variable; over–the-counter medications: variable; formal and informal carers: $34.24/hour^b^.

*p ≤ 0.05. ^a^death weights have been applied to this data set to represent time in the study pertaining to patient death. ^b^TAC [13]. MWU, Mann–Whitney *U* test.

### Health service utilization questionnaire

As the administrative data were limited to acute episodes (including acute phase rehabilitation) for the primary health service and non-hospital health care for Medicare (for example medical and pharmaceuticals), a self-reported health service utilization questionnaire was designed to supplement these data (Additional file 1). The questionnaire was completed for participants at 6 and 12 months following discharge from rehabilitation, administered by assessors blinded to group allocation. Participants reported admissions and length of stay of acute or rehabilitation hospital admissions in facilities outside of the primary health service, as well as primary care. There were no double counting of Medicare and primary health service data in the self-reported health service utilization data because the self-reported data were checked against both the Medicare and primary health service data. Where we had recorded the same services, these were removed from the self-reported data. For example, General Practitioner community visits were reported in both the self-reported and Medicare data, hence they were removed from the self-reported data prior to the final analyses. The number and type of non-Medicare health care visits, over-the-counter medications and the assistance of carers were also collected. Carers included community paid carers (for example a patient care attendant) and informal unpaid carers (for example a family member).

### Costing method

All costs were reported as 2012/2013 Australian dollars (AUD$) (international currency conversion available http://www.xe.com/currencyconverter/#converter [11]). Administrative cost data collected for the primary health service, Medicare and PBS covered three financial years. Inflation rates of 3.7% and 7.2% respectively, were applied to the 2010/2011 and 2011/2012 data consistent with the health care inflation rate of the national Consumer Price Index [12].

Self-reported health service utilization costs for hospital admissions outside of the primary health service were calculated as an average cost per day, based on data from the primary health service for acute and rehabilitation admissions in 2012/2013. Out-of-pocket costs were included in the charges reported in the Medicare data for medical and non-medical services and pharmaceuticals. The cost of non-Medicare health care services was based on charges for community allied health services from the Victorian Transport Accident Commission [13]. Over-the-counter medications were costed according to the actual drug used (Tables 1 and 2) [14]. Informal (unpaid) care was costed using the total hours reported by patients multiplied by the hourly rate for a community health care attendant, to represent the true resource cost of an equivalent formal health care professional.

### Clinical outcomes measures

The EuroQol (EQ-5D-3L) questionnaire [15,16] was used to measure patient health related quality of life and was reported as a utility index score. A MCID in quality of life was based on the recommendation to use half a standard deviation of the baseline score [17]. Applied to the quality of life utility index score in this study, an increase of 0.18 represented a MCID in quality of life [4]. Functional independence was measured using the FIM [18] administered by credentialed assessors. An increase in the FIM score of 22 points (scores range from 18, lowest function, to 126, highest function) was considered to be a MCID in functional independence based on previous literature [19]. Both outcome measures were administered on admission (baseline) and 6 and 12 months following discharge from rehabilitation by assessors blind to group allocation.

### Statistical analysis

Mean cost difference was determined between the two groups using an independent *t*-test at 6 months and at 12 months [20]. Cost data in an economic evaluation is typically positively skewed and this is often due to a small number of patients with high costs, although with samples of greater than 150 participants it is reported that the *t*-test is generally robust [21]. To acknowledge the potential for skewed cost data, we have presented the mean, SD and range for all cost data, as well as the *p*-value for the *t*-test and the non-parametric equivalent, the Mann–Whitney *U* test for total costs at 6 and 12 months. Between group differences in the quality of life utility score and the functional independence score were calculated with analysis of covariance (ANCOVA) using the baseline score as covariate [22,23]. To further address the potential for a skewed distribution of costs and outcomes, relative risks (RRs) were used to calculate the proportion of participants achieving a MCID in the quality of life utility score and the functional independence score at 6 and 12 months following discharge [4]. Analysis was completed using intention-to-treat principles. Missing clinical outcome data and resource use (due to non-consent for the release of Medicare and PBS data or failure to complete the health utilization questionnaire at 6 or 12 months) were managed using a multiple imputation method via chained equations imputation generating 5 imputed datasets [4,24]. Where participants had died (confirmed by the next of kin at the 6 or 12 month survey), they were included in the analyses, with total costs weighted by time in the study. As the exact date of death was unknown, the last date of service from the Medicare data was used as a proxy. For example, if a death was confirmed at the 12 month survey, and the last Medicare service was at 9 months, it was assumed that the person participated in 75% of the follow up period, and costs were weighted at .75. Incident rate ratios (IRR) were analysed for each of the resources in the follow up period to report the difference in event rate between the control and intervention groups.

Incremental cost effectiveness ratios (ICERs) and their associated confidence intervals (CIs) for quality of life utility and functional independence scores were calculated at the sample level using the bootstrap method (5000 repetitions) [25]. Individual ICERs were used to generate confidence ellipses and cost effectiveness acceptability curves (CEACs), using the central limit theorem, to illustrate cost effectiveness across a range of willingness-to-pay values [25]. The use of either the central limit theorem or the bootstrapping method is appropriate to obtain uncertainty around the mean difference when there is a moderate to large sample size (n > 50), even when data are highly skewed [25]. While the ellipses provide important information relating to the statistical significance of the intervention under review, the CEACs allow comparison between different interventions. The ICER for the quality of life utility score represents the cost per QALY gained. The ICER for a one point change in the functional independence score was multiplied to report the cost difference for a 22 point change in the functional independence score, representing the marginal cost for a MCID in functional independence.

Analyses were completed with IBM SPSS Statistics Version 21 [26], STATA Version 12 [27] and the ICER, CI, CI ellipse and CEAC were completed with software in Microsoft Excel (spreadsheet available from Nixon RM, Wonderling D, Grieve RD. 2010) [25]. All statistical tests were conducted at a 5% level of significance and reported with 95% confidence intervals (CI) unless otherwise stated.

### Sensitivity analysis

A number of sensitivity analyses were performed on the total cost at 6 and 12 months. Two assumptions were made for informal care; the first was to reduce the self-reported amount by 50%, the second was to remove it altogether. Fifty percent meant that the hours were capped at 12 hours per day representative of a nursing shift. Removing informal care was to allow comparison with previous literature, where the inclusion of informal care is inconsistent, reported in less than 10% of cost utility analyses [28,29].

Data from the self-reported health service utilization questionnaire were adjusted to address the uncertainty of recall bias. To determine the direction and amount of adjustment, we compared community General Practitioner (GP) visits recorded in the Medicare administrative data to self-reported data for those participants who were alive at 12 months, had completed both the 6 and 12 month questionnaires and for whom we had 12 months of Medicare data. In addition, self-reported health service utilization data were removed so that only administrative data from the primary health service and Medicare were included, to account for unpredictable variability in self reporting.

### Statement of ethics approval

The study obtained ethics approval from Eastern Health Research and Ethics Committee (reference number E58 09/10) and La Trobe University Human Research Ethics Committee (reference number FHEC10/14), including participant consent to meet requirements for Medicare and PBS data. All patients gave informed written consent prior to taking part.

## Results

### Participants

A total of 996 patients were randomized to the control group (n = 500) or the intervention group (n = 496) with the flow of patients through the trial reported elsewhere [4]. Patients had a mean age of 74 years (standard deviation (SD) 13) and 631 (63%) were women. The groups appeared similar for diagnosis and co-morbidities and included 581 (58%) with an orthopedic diagnosis, and 203 (20%) with a neurological diagnosis.

At 12 months, 101 participants had died (intervention group n = 52, control group n = 49) and at 6 months, 65 participants had died (intervention group n = 31, control group n = 34). At 12 months, 82% (n = 812) of the total participants were available for follow up and had completed the health service utilization questionnaire (intervention group n = 401, control group n = 411). Of the 812 participants, there were 460 living at home independently (intervention group n = 231, control group n = 229) and a further 229 living at home with the support of formal or informal carers (intervention group n = 114, control group n = 115).

Values were imputed for the missing data from the 83 participants (8%) who were alive and did not complete the health service questionnaire at 12 months, and for the 52 participants who did not consent to Medicare and PBS data (5%). Administrative data were available from the primary health service for all participants (100%) for the period between the initial rehabilitation admission and 12 months following discharge from rehabilitation.

### Cost and utilization data

The mean total cost for the initial rehabilitation admission and the 12 months following discharge from rehabilitation was $61,859 (SD 79,798) for the intervention group and $68,184 (SD 82,922) for the control group, with a mean cost difference of -$6,325 (95% CI −16,730 to 4,081; *t* test *p* = 0.23 and *MWU p* = 0.06) in favor of the intervention group (Table 1). The mean total cost for the initial rehabilitation admission and the 6 months following discharge from rehabilitation was $37,170 (SD 44,351) for the intervention group and $43,615 (SD 49,806) for the control group, with a mean cost difference of -$6,445 (95% CI −9,522 to −3,368; *t test p* = 0.04 and *MWU p* = 0.01) in favor of the intervention group (Table 2).

Mean hospital length of stay for all admissions to the primary health service during the 12 months following discharge from rehabilitation was 10.3 days (SD 24.5; n = 496) for the intervention group and 15.2 days (SD 37.4; n = 500) for the control group, with a mean difference of −4.9 days (95% CI −9.0 to −0.9; p = 0.02) in favor of the intervention group (Table 1). The intervention group had an observed event rate of 29% (IRR 0.71; 95% CI 0.52 to 0.96) less than the control group for the risk of readmission to either the primary health service or to another health service over the 12 months following discharge, and an event rate of 34% (IRR 0.66; 95% CI 0.45 to 0.98) less than the control group over 6 months following discharge. Cost and utilization data relating to other health care services, Medicare, PBS and self-reported health utilization data included in the economic evaluation are reported in Tables 1 and 2. For most individual health services there was a pattern of less resource utilization and less cost that favored the intervention group, although these differences were not statistically significant. Excluding readmission to either the primary health service or to another health service, all other health services had a non-significant difference in the observed event rate between the control and the intervention groups, for the 6 and 12 month periods following discharge.

### Clinical outcomes measures

Difference between the intervention and control groups for the health related quality of life utility index score was non-significant between admission and 12 months following discharge from rehabilitation (MD 0.01, 95% CI −0.04 to 0.05; p = 0.77) and at 6 months following discharge (MD 0.03, 95% CI −0.01 to 0.08; p = 0.15) (Table 3). At 12 months, participants in the intervention group were 11% more likely to achieve a MCID in health related quality of life utility index score (RR 1.11; 95% CI 1.00 to 1.24) and at 6 months they were 19% more likely to do so (RR 1.19; 95% CI 1.06 to 1.34), compared to the control group. The difference between the intervention and control groups for the functional independence score was non-significant between admission and 12 months following discharge from rehabilitation (MD 1.3, 95% CI −0.9 to 3.5; p = 0.24), although the intervention group had a higher positive change between admission and 6 months following discharge (MD 2.0, 95% CI 0.0 to 4.0; p = 0.05) (Table 3). Participants in the intervention group were not more likely to achieve a MCID in their functional independence score at either 12 months (RR 1.07; 95% CI 0.96 to 1.20) or at 6 months following discharge (RR 1.06; 95% CI 0.94 to 1.19) compared to the control group.

**Table 3:** **Outcome measures on admission, 6 and 12 months**

	**Groups**						**Difference between groups**	
	**Admission**		**Month 6 following discharge**		**Month 12 following discharge**		**Month 6**	**Month 12**
	**Mean (SD; range)**		**Mean (SD; range)**		**Mean (SD; range)**			
	**Intervention**	**Control**	**Intervention**	**Control**	**Intervention**	**Control**	**Intervention - Control**	**Intervention - Control**
	**(n = 496)**	**(n = 500)**	**(n = 496)**	**(n = 500)**	**(n = 496)**	**(n = 500)**		
FIM total	84	84	109	107	109	108	2.0	1.3
	(19; 19 to 119)	(20; 0 to 124)	(17; 22 to 126)	(19; 21 to 126)	(17; 19 to 126)	(19; 21 to 126)	(0.0 to 4.0)	(−0.9 to 3.5)
							p = 0.05*	p = 0.24
EQ-5D-3L utility index	0.32	0.37	0.63	0.61	0.64	0.64	0.03	0.01
	(0.35; −0.59 to 1.00)	(0.35; −0.59 to 1.00)	(0.36; −0.35 to 1.00)	(0.37; −0.43 to 1.00)	(0.39; −0.59 to 1.00)	(0.34; −0.59 to 1.00)	(−0.01 to 0.08)	(−0.04 to 0.05)
							p = 0.15	p = 0.77

NB: Intervention = Monday to Saturday rehabilitation, Control = Monday to Friday rehabilitation, EQ-5D-3L utility index = EuroQOL questionnaire, VAS = Visual Analogue Scale. *p ≤ 0.05.

### Incremental cost effectiveness ratio

In summary, the results indicate that the intervention is associated with lower costs and improved health outcomes. The ICER and associated 95% CIs may be interpreted as follows; each of the ICERs are positive which represents a cost saving to the health system. When the lower value of the 95% CI is negative, this represents a potential additional cost, and when the lower value of the 95% CI is positive, this represents a potential cost saving. Each of the upper values of the reported 95% CIs are positive which represents a potential cost saving. The ICER for the initial rehabilitation admission and the 12 months following discharge from rehabilitation reported a non-significant cost saving of $282,144 (95% CI −1,074,914 to 1,520,885) per QALY gained for the intervention group compared to the control group. The ICER for this same period reported a non-significant cost saving of $10,199 (95% CI −63,526 to 81,511) for a one point change in the functional independence score for the intervention group compared to the control group; or a cost saving of $200,797 (95% CI −1,300,355 to 1,572,604) for a MCID in functional independence for the intervention group compared to the control group.

The ICER was statistically significant at 6 months following discharge showing a cost saving of $112,320 (95% CI 6,556 to 336,631) per QALY gained for the intervention group compared to the control group. The ICER for this same period reported a non-significant cost saving of $2,540 (95% CI −14,972 to 24,597) for a one point change in the functional independence score for the intervention group compared to the control group, or a non-significant cost saving of $99,580 (95% CI −254,401 to 543,098) for a MCID in functional independence for the intervention group compared to the control group.

**Figure 1:**
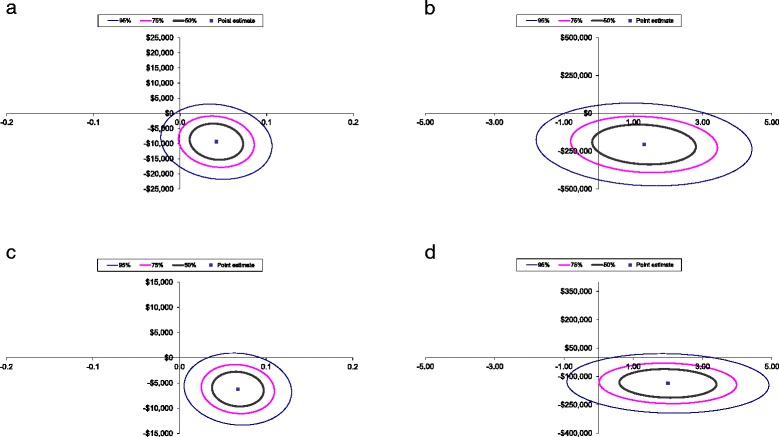
**a**-**d** Confidence ellipses for intervention versus control for the incremental cost (vertical axis AUD$2012/13) per incremental outcome gained (horizontal axis). (**a**) Inpatient admission and 12 months following discharge for incremental cost per quality adjusted life year (QALY) gained. (**b**) Inpatient admission and 12 months following discharge for incremental cost per minimal clinically important difference (MCID) in function. (**c**) Inpatient admission and 6 months following discharge for incremental cost per quality adjusted life year (QALY) gained. (**d**) Inpatient admission and 6 months following discharge for incremental cost per minimal clinically important difference (MCID) in function.

The ICER point estimation and the confidence interval ellipses (50%, 75% and 95%) for the 6 and 12 months following discharge from rehabilitation are presented in Figure 1. The confidence ellipses show that most of the 50%, 75% and 95% confidence intervals sit within the bottom right quadrant of the cost effectiveness plane and only a small portion falls in the upper right or lower left quadrants [30].

### Cost effectiveness acceptability curve

**Figure 2:**
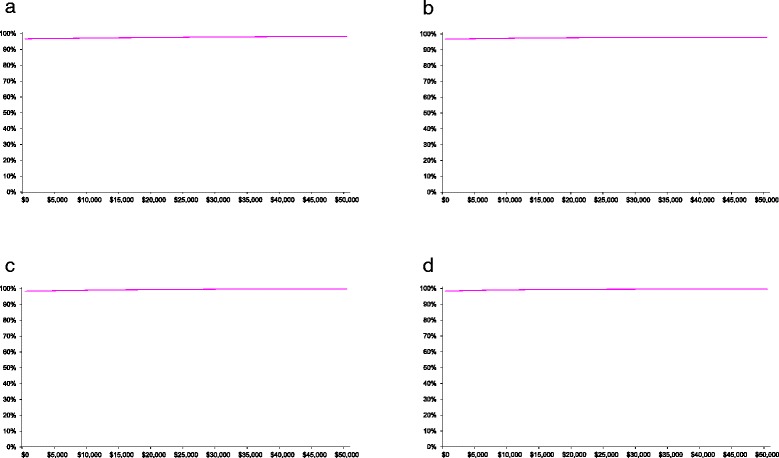
**a**-**d** Cost effectiveness acceptability curve for intervention versus control for the probability of cost effectiveness (vertical axis) versus a range of cost effectiveness willingness to pay values (AUD$2012/13) per incremental outcome gained (horizontal axis). (**a**) Inpatient admission and 12 months for incremental cost per quality adjusted life year (QALY) gained. (**b**) Inpatient admission and 12 months for incremental cost per minimal clinically important difference (MCID) in function. (**c**) Inpatient admission and 6 months for incremental cost per quality adjusted life year (QALY) gained. (**d**) Inpatient admission and 6 months for incremental cost per minimal clinically important difference (MCID) in function.

For the initial rehabilitation admission and in the 12 months following discharge from rehabilitation, the curves were relatively flat. For a willingness to pay of $50,000 there was 98% certainty of cost effectiveness for a QALY gained or for a MCID in functional independence (Figure 2). Alternatively for this same time period, for a willingness to pay of zero dollars, there was 97% certainty of cost effectiveness for a QALY gained or for a MCID gained in functional independence.

For the initial rehabilitation admission and in the 6 months following discharge from rehabilitation the curves were relatively flat. For a willingness to pay of $23,000 there was almost 100% certainty of cost effectiveness for a QALY gained or for a MCID gained in functional independence (Figure 2). Alternatively for this same time period, for a willingness to pay of zero dollars, there was 98% certainty of cost effectiveness for a QALY gained or for a MCID gained in functional independence.

### Sensitivity analyses

Reducing the amount of informal care by 50% over the 12 months following discharge from rehabilitation resulted in a non-significant mean cost difference of -$5,518 (95% CI −12,155 to 1,119; p = 0.10) in favor of the intervention group. Reducing the amount to zero resulted in a significant mean cost difference of -$4,711 (95% CI −9,132 to −290; p = 0.04) in favor of the intervention group (Table 4).

**Table 4:** **Sensitivity analysis for the total costs between the initial inpatient admission and 6 or 12 months following discharge (AUD$2012/13)**

	**Mean cost per patient for the period between the initial inpatient admission and 6 or 12 months following discharge (SD; range)** ^**a**^ **$2012/13**	**Difference between groups for costs**	
	**Intervention**	**Control**	**Intervention minus control (95% CI)**
	**(n = 496)**	**(n = 500)**	
**12 months**			
Mean total cost base case	61,859	68,184	−6,325
	(79,798; 4,845 to 463,656)	(82,922; 4,845 to 463,656)	(−16,730 to 4,081) p = 0.234
Informal care reduced by 50%	47,774	53,292	−5,518
	(49,820; 4,656 to 271,517)	(53,891; 4,845 to 279,795)	(−12,155 to 1,119) p = 0.103
Informal care reduced to 0	33,689	38,400	−4,711
	(31,704; 4,656 to 222,674)	(37,145; 4,845 to 225,707)	(−9,132 to −290) p = 0.037*
Health service utilization data increased by 15%	66,788	73,448	−6,660
	(89,786; 4,678 to 458,171)	(92,978; 4,862 to 522,237)	(−18,346 to 5,026) p = 0.264
Health service utilization data reduced to 0	29,001	33,089	−4,089
	(28,812; 2,652 to 222,805)	(34,562; 4,246 to 225,016)	(−6,165 to – 2,012) p = 0.049*
**6 months**			
Mean total cost base case	37,170	43,615	−6,445
	(44,351; 3,713 to 231,733)	(49,806; 3,723 to 256,182)	(−9,522 to −3,368) p = 0.036*
Informal care reduced by 50%	30,533	35,698	−5,165
	(30,043; 3,713 to 193,317)	(35,204; 3,723 to 187,463)	(−7,301 to −3,030) p = 0.016*
Informal care reduced to 0	23,896	27,782	−3,886
	(22,186; 3,713 to 183,970)	(27,729; 3,723 to 184,894)	(−5,641 to −2,131) p = 0.018*
Health service utilization data increased by 15%	39,569	46,464	−6,894
	(49,366; 3,713 to 254,588)	(55,146; 3,713 to 285,570)	(−10,309 to −3,480) p = 0.043*
Health service utilization data reduced to 0	21,175	24,621	−3,446
	(20,203; 2,652 to 179,677)	(26,116; 2,638 to 184,543)	(−4,970 to −1,922) p = 0.024*

*p ≤ 0.05. ^a^death weights have been applied to this data set to represent time in the study pertaining to patient death.

Of the 752 participants with complete data at 12 months, there were 9,419 self-reported community GP visits compared to 10,855 visits recorded by Medicare for claims for GP services. This suggests under reporting of self-reported primary health care services. Based on these data, the number of self-reported health services utilized in the 12 months following discharge from rehabilitation was inflated by 15%. This resulted in a non-significant mean cost difference of -$6,660 (95% CI −18,346 to 5,026; p = 0.26) in favor of the intervention group; reducing the amount to zero resulted in a significant mean cost difference of -$4,089 (95% CI −6,165 to −2,012; p = 0.05) in favor of the intervention group (Table 4).

The sensitivity analyses were repeated for the period between the initial rehabilitation admission and in the 6 months following discharge with similar findings that all significantly favored the intervention group (Table 4).

## Discussion

From a health system perspective inclusive of private costs, the provision of a Saturday rehabilitation service to inpatients in addition to Monday to Friday, improved QALYs as well as functional status, and reduced costs compared with usual Monday to Friday care at both 6 and 12 months following discharge from rehabilitation.

One explanation for why the intervention was cost effective at 6 months and possibly cost effective at 12 months was that participants who received the additional Saturday rehabilitation were discharged at a higher level of functional independence [4] and therefore were equipped to live independently in the community with less dependence on community health services. An interpretation is that the quantity of inpatient rehabilitation does matter; for the patient population in our trial most functional gains occurred during the inpatient stay, with minimal gains following discharge. For example in the current trial, participants in the intervention group had significant gains in their FIM score from admission to discharge (26%), with lesser gains from discharge to 6 months (2%) and no change from 6 to 12 months [4]. These results support previous studies that report most functional gains occur in the initial rehabilitation period and after this functional status largely remains the same [31,32]. Health service resource and cost data that favored the intervention group were realized between admission and 6 months (p = 0.04) but not between 6 and 12 months following discharge.

During the initial inpatient rehabilitation admission there was a potential cost saving from a reduction in length of stay. However, this needs to be considered in the context of the direct additional costs to run a Saturday rehabilitation program [3]. The additional resources required for a Saturday service include therapist time, space, equipment and consumable items. On average these additional resource costs for providing the additional Saturday rehabilitation service were less than $200 per admission, in comparison with the overall observed saving of over $1,600 per admission [3]. This demonstrates that the additional costs of running the Saturday service were offset by a substantial saving over the duration of the rehabilitation admission.

From admission to 12 months following discharge most of the savings to the health system occurred from the reduction in hospital readmissions, with no significant differences in community care services, including medical services. There was no observed cost shift into the community; in fact there was an ongoing cost saving to the community that included reduced readmissions to the primary and other health services. A cost shift may involve reporting false “savings” based on redistributing the cost from one health service into the broader community health systems [33]; this would be evident if the intervention group had reduced health service utilization in the inpatient stay, but had higher health service utilization across any or all of the other health services in the 12 months following discharge. This was not the case. The intervention group had reduced health service utilization during their inpatient stay with ongoing reduced health service utilization in the 12 months following discharge. These results are consistent with other inpatient rehabilitation intervention studies that report that cost savings during the initial rehabilitation period are associated with a reduced length of stay, and with no cost shift following discharge [8,34].

Another example of cost shift is the reliance on informal care once the patient is discharged from the rehabilitation facility. That is, the cost of care is shifted from the health system into the household and it is frequently family and friends who provide the unpaid care. In addition to this being reported as a potential cost shift, there needs to be consideration of the burden that this may place on the household [35]. In Australia, it is estimated that the annual cost of replacing informal care with paid care is $4.8 billion [36]. Despite this substantial cost to society, support for the inclusion of informal care in an economic evaluation, is not consistent [28,29,37,38]. The scope of this economic evaluation included the utilization of informal care which represented 43% of the total costs. Sixteen percent of our participants had survived a stroke and the cost of informal care was within the range of costs reported by other studies with a stroke cohort where informal care represented 4% to 55% of the total costs [7,8,39]. Contributing to this variation could be different methods for costing informal care, such as allotting one-third the cost of the average national wage [39], price equivalence to the cost of hostel care [7], the minimum wage [8], the cost of a home help worker [8] or our method of uncapped actual daily hours valued at that of an equivalent paid formal carer. The variation in including the cost of informal care, together with the variation of assumptions of costing of informal care, supported our sensitivity analyses that varied, and removed the cost of informal care. When the amount of informal care was reduced to zero, the observed difference in total cost at 12 months following discharge between the intervention and control groups was significant and in favor of the intervention group.

This economic evaluation has implications for both health service managers, and policy makers. The provision of an additional Saturday inpatient rehabilitation service provided benefits that reduced short term costs to the health service and reduced the medium term costs across the health system including a reduction in hospital readmissions that could potentially free up inpatient beds [40]. In support of this, participants who received additional Saturday rehabilitation had a mean reduction of 4.9 days (95% CI −9.0 to −0.9; p = 0.02; IRR 0.71; 95% CI 0.52 to 0.96) in admission to hospital in the 12 months following discharge from rehabilitation compared with the control group. The implication is a win-win intervention for the health service, the health system and the patient. In Australia, health care is funded by the government at both national and state levels, as well as by private insurers. At the national level, primary health care is publically funded by Medicare and this includes community based health care services (e.g. a general practitioner or medical specialist), whereas the state government is responsible for the provision of public hospital services, such as the rehabilitation inpatient admissions included in this clinical trial [41]. Funding for public hospital acute and rehabilitation services is via the Australian government and based on a single episode of care; as such readmissions that occur in the 12 month follow up period after the initial rehabilitation hospital episode in this clinical trial are funded as a separate episode of care. Therefore, under output based funding public hospitals have an incentive to discharge acute (including acute phase rehabilitation) patients as early as possible. This contrasts with the private system where private insurance funding may be a per diem amount or a fixed amount for the episode. Theoretically, as the results of our trial show a reduction in total cost, both public and private hospitals should be willing to implement weekend acute rehabilitation services. However, cost savings may be realised at the broader hospital level while the spending and staffing decisions may be realised at the department level, so without resource reallocation and collaboration, the provision of a weekend service may be difficult to implement.

The strengths of this economic evaluation are that it was completed alongside a blinded, fully powered randomized controlled trial and it used an appropriate alternative intervention, as only 30% of Australian rehabilitation inpatient health services offer a weekend physiotherapy service [42]. Other strengths included access to complete clinical cost data on all patients across the primary health service, Medicare and PBS administrative data as well as the inclusion of informal care. A range of rehabilitation diagnoses and patients with a language other than English as their first language were included in the trial representing a general rehabilitation population. A limitation of using a self-reported health service utilization tool is recall bias; however, the sensitivity analysis addressed this by varying the value of this self-reported data. The wide and uneven distribution of the CIs around the ICER are difficult to interpret [43,44], however they have been reported as the sample size is large [25] and the non-parametric bootstrap method was used [43]. The CI ellipses for the ICERs show that most of the 75% and 95% CIs fall within the bottom right hand quadrant. This suggests that the intervention is dominant over the comparator of usual care. This study did not include the wider economic impact from a societal perspective including factors such as return to work, the costs of travel or the cost of nursing home placement. The impact on return to work for this study has been reported elsewhere [6]. Therefore, we are confident that the results are generalizable across public acute phase inpatient rehabilitation settings. There were minor variations to the trial protocol. These included the use of multiple imputation rather than the carry forward technique for missing data, consistent with recent recommendations [24], as well as a reduced data collection period due to a higher than expected rate of participant recruitment. Future research may focus on translation research designed for successful and sustained implementation of this additional Saturday rehabilitation service.

## Conclusion

From a health system perspective inclusive of private costs and informal care, the provision of a rehabilitation service to inpatients on a Saturday in addition to Monday to Friday compared to Monday to Friday rehabilitation alone, is likely to have sustained cost savings per QALY gained and for a MCID in functional independence during the inpatient stay and up to 12 months following discharge from rehabilitation.

## Additional file

Additional file 1:
**6 and 12 Month Follow up Questionnaire.**

## Abbreviations

ANCOVA: Analysis of covariance
AUD$: Australian dollars
CEAC: Cost effectiveness acceptability curve
CHEERS: Consolidated health economic evaluation reporting standards
CI: Confidence interval
DRG: Diagnosis related group
EQ-5D-3 L: EuroQol
GP: FIM, Functional independence measure
GP: General practitioner
ICER: Incremental cost effectiveness ratio
IRR: Incident rate ratios
MCID: Minimal clinical important difference
MD: Mean difference
MWU: Mann–Whitney *U* test
PBS: Pharmaceutical benefit scheme
QALY: Quality adjusted life year
RR: Relative risk
SD: Standard deviation
VAS: Visual analogue scale
