# Supplementary material for: Is cost effectiveness sustained after weekend inpatient rehabilitation? 12 month follow up from a randomized controlled trial
Source: BMC Health Serv Res. 2015 Apr 18;15:165. doi: 10.1186/s12913-015-0822-3 (PMC4438580; doi:10.1186/s12913-015-0822-3)
Supplement: Additional file 1: — 6 and 12 Month Follow up Questionnaire. [file 12913_2015_822_MOESM1_ESM.doc]

**SIX AND TWELVE MONTH FOLLOW UP PHONE CALLS**

| **Research project ID Number:** |  |
| --- | --- |

| **Staff** | **Patient Information for the Follow Up Phone Call Project Officer** | | | | | | | | | | |
| --- | --- | --- | --- | --- | --- | --- | --- | --- | --- | --- | --- |
| **Project Officer** | | Study ID No. |  | | --- | --- | | Month / year of 6 Months follow up due |  | | Month / year of 12 Months follow up due |  | | Campus |  | | First Name |  | | Surname |  | | EH UR No. |  | | DOB |  | | Age |  | | Home Ph. No. |  | | Mobile No. |  | | NOK Name |  | | NOK Ph. No. |  | | Language |  | | Gender |  | | Diagnosis |  | | Admission Date |  | | Discharge Date |  | | Accom Pre-Admission |  | | Accom Post Discharge |  | | Adverse Events |  | | Type - Adverse Events |  | | Follow Up Rehab Services |  | | | | | | | | Points to note for the 6 or 12 months follow up phone calls (e.g. at 6 month phone call patient relocated from home to care facility or patient passed away): | | | |
| **Staff** | | **6/12 post admission – Date completed ___/____/____** | | | | | **12/12 post discharge – Date completed ___/____/____** | | | | |
| **Project Officer (by telephone)** | Follow up questionnaire completed (attach separate sheet) Y/N | | | | | Medicare data collected (separate sheet – to be completed by Health Economist) Y/N | | | | | |
|  |  |  |  |  |  | Follow up questionnaire completed (attach separate sheet) Y/N | | | | | |
|  | EuroQOL: Total Score Part 1:  Individual Score Part 1 Question 1:  Individual Score Part 1 Question 2:  Individual Score Part 1 Question 3:  Individual Score Part 1 Question 4:  Individual Score Part 1 Question 5:  Score Part 2 (out of 100): Comments: | | | | | EuroQOL: Total Score Part 1:  Individual Score Part 1 Question 1:  Individual Score Part 1 Question 2:  Individual Score Part 1 Question 3:  Individual Score Part 1 Question 4:  Individual Score Part 1 Question 5:  Score Part 2 (out of 100): Comments: | | | | | |
|  | FIM Score - Total:  Motor subtotal (items 1 – 13 inclusive):  Individual scores 1:  2:  3: | | 4:  5:  6:  7:  8: | 9:  10:  11:  12:  13: | 14:  15:  16:  17:  18: | FIM Score - Total:  Motor subtotal (items 1 – 13 inclusive):  Individual scores 1:  2:  3: | | | 4:  5:  6:  7:  8: | 9:  10:  11:  12:  13: | 14:  15:  16:  17:  18: |

**PATIENT EuroQOL – 6 MONTHS**

*EuroQOL tool*

*inserted here*

**PATIENT FIM – 6 MONTHS**

*FIM tool*

*inserted here*

**PATIENT HEALTH SERVICES UTILISATION QUESTIONNAIRE FOR 6 MONTHS – Page 1 of 4**

Date: _ _/_ _/_ _ Patient Id No. _ _ _­­­_________ Assessor Id No: Sarah (1) / Rhonda (2)

**1. Person completing the questionnaire:**

□ Patient □ Next of kin □ Main Care Giver □ Other (specify) ________

**2. Current patient location and status:**

□ At home independent □ At home with care □ Low level residential care facility

□ High level residential care facility □ Other (specify) ___________

□ Deceased - Date__/__/__No further questions (End of Questionnaire)

**3. Prior to your rehabilitation at Eastern Health, were you in paid employment?** □ No (i.e. retired prior to rehab) □ Yes

Please indicate the position that most describes your employment status prior to you rehabilitation at Eastern Health:

| ***Paid*** | ***Unpaid*** | ***Unable to work*** |
| --- | --- | --- |
| □ Employment:  Average hours per week _____ | □ Study:  Average hours per week _____ | □ Unemployed |
| □ Current on the job training:  Average hours per week _____ | □ Volunteer work:  Average hours per week _____ | □ Unfit / unable to work |
| □ Sheltered workshop:  Average hours per week _____ | □ Home maker:  Average hours per week _____ | □ Forced retirement due to medical condition associated with rehabilitation stay |
| □ Retired since rehabilitation hospital stay | □ Other (specify) ___________:  Average hours per week _____ | □ Need further training / rehabilitation prior to employment |

**4. If YES to paid employment ask type:** Employee / Independent Contractor / Business Operator

Industry (see list): _____________________________

Occupation (see list): __________________________

**5. Now that it is 6 months since your rehabilitation at Eastern Health, are you currently in paid employment?** □ No □ Yes

Please indicate the position that most describes your employment status at the moment, now that it is 6 months since rehabilitation:

| ***Paid*** | ***Unpaid*** | ***Unable to work*** |
| --- | --- | --- |
| □ Employment:  Average hours per week _____ | □ Study:  Average hours per week _____ | □ Unemployed |
| □ Current on the job training:  Average hours per week _____ | □ Volunteer work:  Average hours per week _____ | □ Unfit / unable to work |
| □ Sheltered workshop:  Average hours per week _____ | □ Home maker:  Average hours per week _____ | □ Forced retirement due to medical condition associated with rehabilitation stay |
| □ Retired since rehabilitation hospital stay | □ Other (specify) ___________:  Average hours per week _____ | □ Need further training / rehabilitation prior to employment |

**6. If YES to paid employment ask type:** Employee / Independent Contractor / Business Operator

Industry (see list): _____________________________

Occupation (see list): __________________________

**7. If YES to currently in paid employment, since your rehabilitation have you had to reduce the number of hours per week you work over the last 6 months?** Confirm reason / details below (Please tick only one)

□ No, I work the same hours.

□ Yes, I have had to reduce my hours per week by ……………….(current hours per week)

□ Yes, I have had to stop work completely

**PATIENT HEALTH SERVICES UTILISATION QUESTIONNAIRE FOR 6 MONTHS – Page 2 of 4**

**8. Do you require regular assistance from family members or friends (carers) as a result of your condition?** □ No □ Yes

If Yes, please state how many family/friends carers you have in total ………………

Please state the relationship of your main carer:……………………………………….

In the last 6 months since your rehabilitation, please state how many hours on average each carer has spent caring for you per week:

Main carer: …………..hours per week Other carer 1:……….hours per week

Other carer 2: ………..hours per week Other carer 3:……….hours per week

Does your main carer receive a carer’s payment or allowance? □ No □ Yes

**9. Are you currently receiving benefits?** □ No □ Yes

If Yes, what benefit have you been receiving in the last 6 months? (tick all that apply)

□ Disability Support Pension □ Aged Pension

□ Sickness Allowance □ Widow Allowance

□ Unemployment (‘New Start’) Allowance □ Mature Age Allowance

□ Mobility Allowance □ Partner Allowance

□ Other ……………

**10. Healthcare Visits:**

a) Since your discharge from the rehabilitation hospital 6 months ago have you needed to visit any of the following health professionals:

|  | ***No*** | ***Yes*** | ***Number of times*** |
| --- | --- | --- | --- |
| General Practitioner (GP) |  |  |  |
| Medical Specialist |  |  |  |
| Physiotherapist |  |  |  |
| Occupational Therapist |  |  |  |
| Other Allied Health: Specify ________________ |  |  |  |
| Other Allied Health: Specify ________________ |  |  |  |
| Community or District Nurse |  |  |  |
| X-Ray Clinic |  |  |  |
| Pathology Clinic |  |  |  |
| Hospital Emergency Department |  |  |  |
| Hospital Outpatient Clinic |  |  |  |
| Other: Specify ____________________ |  |  |  |
| Other: Specify ____________________ |  |  |  |
| Other: Specify ____________________ |  |  |  |

**PATIENT HEALTH SERVICES UTILISATION QUESTIONNAIRE FOR 6 MONTHS – Page 3 of 4**

**11. Since your discharge from the rehabilitation hospital 6 months ago have you been admitted to a general hospital?:**

□ No □ Yes, If yes,

How many times were you admitted to hospital? _______________

How many nights did you stay in hospital? ____________________nights

In which month were you admitted to hospital? _________________

What was the name of the hospital? _________________________

Was your hospital in the Eastern Health Network? □Yes □No

**12. Since your discharge from the rehabilitation hospital 6 months ago have you been admitted to a rehabilitation hospital?:**

□ No □ Yes, If yes,

How many times were you admitted to hospital? ___________

How many nights did you stay in hospital? ____________________nights

In which month were you admitted to hospital? ________________

What was the name of the hospital? _________________________

Was your hospital in the Eastern Health Network? □Yes □No

Was it the same rehabilitation hospital as before? □Yes □No

**PATIENT HEALTH SERVICES UTILISATION QUESTIONNAIRE FOR 6 MONTHS – Page 4 of 4**

**13. Medication use:**

List your current medications and how many times you take them each day:

- Check with the list of medications given to the patient on discharge from in-patient rehabilitation – if they still have this
- Ask if the patient is taking any "over the counter" medications (e.g. pain relief - panadol / topical anti-inflammatory / patches / herbal / other)
- Ask if the patient is taking any mediations from a private prescription source (e.g. methadone / pain program)

| **Medication Name** | **Trade Brands** | **Usual dosage** | **Indication** | **Tick if this medication is taken by the patient** | **Dose strength (e.g. 500mg in each tablet)** | **Dose quantity (e.g. 2 Tablets)** | **Daily Frequency** |
| --- | --- | --- | --- | --- | --- | --- | --- |
|  |  |  |  |  |  |  |  |
|  |  |  |  |  |  |  |  |
|  |  |  |  |  |  |  |  |
|  |  |  |  |  |  |  |  |
|  |  |  |  |  |  |  |  |
|  |  |  |  |  |  |  |  |
|  |  |  |  |  |  |  |  |
|  |  |  |  |  |  |  |  |
|  |  |  |  |  |  |  |  |
|  |  |  |  |  |  |  |  |
|  |  |  |  |  |  |  |  |
|  |  |  |  |  |  |  |  |
|  |  |  |  |  |  |  |  |
|  |  |  |  |  |  |  |  |
|  |  |  |  |  |  |  |  |
|  |  |  |  |  |  |  |  |
|  |  |  |  |  |  |  |  |
|  |  |  |  |  |  |  |  |
|  |  |  |  |  |  |  |  |
|  |  |  |  |  |  |  |  |
|  |  |  |  |  |  |  |  |
|  |  |  |  |  |  |  |  |
|  |  |  |  |  |  |  |  |
|  |  |  |  |  |  |  |  |
|  |  |  |  |  |  |  |  |
|  |  |  |  |  |  |  |  |
|  |  |  |  |  |  |  |  |

* Attach a separate sheet if more space is required to list the current patient medications

**14. Other comments initiated by the patient:**

**PATIENT EuroQOL – 12 MONTHS**

*EuroQOL tool*

*inserted here*

**PATIENT FIM – 12 MONTHS**

*FIM tool*

*inserted here*

**PATIENT HEALTH SERVICES UTILISATION QUESTIONNAIRE FOR 12 MONTHS – Page 1 of 3**

Date: _ _/_ _/_ _ Patient Id No. _ _ _ Assessor Id No: _ _ _

**1. Person completing the questionnaire:**

□ Patient □ Next of kin □ Main Care Giver □ Other (specify) ________

**2. Current patient location and status:**

□ At home independent □ At home with care □ Low level residential care facility

□ High level residential care facility □ Other (specify) ___________

□ Deceased - Date__/__/__No further questions (End of Questionnaire)

**3. Now that it is 12 months since your rehabilitation at Eastern Health, are you currently in paid employment?** □ No □ Yes

Please indicate the position that most describes your employment status at the moment, now that it is 12 months since rehabilitaiton:

| ***Paid*** | ***Unpaid*** | ***Unable to work*** |
| --- | --- | --- |
| □ Employment:  Average hours per week _____ | □ Study:  Average hours per week _____ | □ Unemployed |
| □ Current on the job training:  Average hours per week _____ | □ Volunteer work:  Average hours per week _____ | □ Unfit / unable to work |
| □ Sheltered workshop:  Average hours per week _____ | □ Home maker:  Average hours per week _____ | □ Forced retirement due to medical condition associated with rehabilitation stay |
| □ Retired since rehabilitation hospital stay | □ Other (specify) ___________:  Average hours per week _____ | □ Need further training / rehabilitation prior to employment |

**4. If YES to paid employment ask type:** Employee / Independent Contractor / Business Operator

Industry (see list): _____________________________

Occupation (see list): __________________________

**5. If YES to currently in paid employment, since your rehabilitation have you had to reduce the number of hours per week you work over the last 12 months?** Confirm reason / details below (Please tick only one)

□ No, I work the same hours.

□ Yes, I have had to reduce my hours per week by ……………….……………….(current hours per week)

□ Yes, I have had to stop work completely

**6. Do you require regular assistance from family members or friends (carers) as a result of your condition?** □ No □ Yes

If Yes, please state how many family/friends carers you have in total ………………

Please state the relationship of your main carer:……………………………………….

In the last 6 months since the previous questionnaire, please state how many hours on average each carer has spent caring for you per week:

Main carer: …………..hours per week Other carer 1:……….hours per week

Other carer 2: ………..hours per week Other carer 3:……….hours per week

Does your main carer receive a carer’s payment or allowance? □ No □ Yes

**PATIENT HEALTH SERVICES UTILISATION QUESTIONNAIRE FOR 12 MONTHS – Page 2 of 3**

**7. Are you currently receiving benefits?** □ No □ Yes

If Yes, what benefit have you been receiving in the last 6 months? (tick all that apply)

□ Disability Support Pension □ Aged Pension

□ Sickness Allowance □ Widow Allowance

□ Unemployment (‘New Start’) Allowance □ Mature Age Allowance

□ Mobility Allowance □ Partner Allowance

□ Other ……………

**8. Healthcare Visits:**

a) In the last 6 months (since you completed the last questionnaire) have you needed to visit any of the following health professionals:

|  | ***No*** | ***Yes*** | ***Number of times*** |
| --- | --- | --- | --- |
| General Practitioner (GP) |  |  |  |
| Medical Specialist |  |  |  |
| Physiotherapist |  |  |  |
| Occupational Therapist |  |  |  |
| Other Allied Health: Specify ________________ |  |  |  |
| Other Allied Health: Specify ________________ |  |  |  |
| Community or District Nurse |  |  |  |
| X-Ray Clinic |  |  |  |
| Pathology Clinic |  |  |  |
| Hospital Emergency Department |  |  |  |
| Hospital Outpatient Clinic |  |  |  |
| Other: Specify ____________________ |  |  |  |
| Other: Specify ____________________ |  |  |  |
| Other: Specify ____________________ |  |  |  |

**9. In the last 6 months (since you completed the last questionnaire) have you been admitted to a general hospital?:**

□ No □ Yes, If yes,

How many times were you admitted to hospital? ___________

How many nights did you stay in hospital? ____________________nights

In which month were you admitted to hospital? _________________

What was the name of the hospital? _________________________

Was your hospital in the Eastern Health Network? □Yes □No

**10. In the last 6 months (since you completed the last questionnaire) have you been admitted to a rehabilitation hospital?:**

□ No □ Yes, If yes,

How many times were you admitted to hospital? ___________

How many nights did you stay in hospital? ____________________nights

In which month were you admitted to hospital? ________________

What was the name of the hospital? _________________________

Was your hospital in the Eastern Health Network? □Yes □No

Was it the same rehabilitation hospital as before? □Yes □No

**PATIENT HEALTH SERVICES UTILISATION QUESTIONNAIRE FOR 12 MONTHS – Page 3 of 3**

**11. Medication use:**

List your current medications and how many times you take them each day:

- Check with the list of medications given to the patient on discharge from in-patient rehabilitation – if they still have this
- Ask if the patient is taking any "over the counter" medications (e.g. pain relief - panadol / topical anti-inflammatory / patches / herbal / other)
- Ask if the patient is taking any mediations from a private prescription source (e.g. methadone / pain program)

| **Medication Name** | **Trade Brands** | **Usual dosage** | **Indication** | **Tick if this medication is taken by the patient** | **Dose strength (e.g. 500mg in each tablet)** | **Dose quantity (e.g. 2 Tablets)** | **Daily Frequency** |
| --- | --- | --- | --- | --- | --- | --- | --- |
|  |  |  |  |  |  |  |  |
|  |  |  |  |  |  |  |  |
|  |  |  |  |  |  |  |  |
|  |  |  |  |  |  |  |  |
|  |  |  |  |  |  |  |  |
|  |  |  |  |  |  |  |  |
|  |  |  |  |  |  |  |  |
|  |  |  |  |  |  |  |  |
|  |  |  |  |  |  |  |  |
|  |  |  |  |  |  |  |  |
|  |  |  |  |  |  |  |  |
|  |  |  |  |  |  |  |  |
|  |  |  |  |  |  |  |  |
|  |  |  |  |  |  |  |  |
|  |  |  |  |  |  |  |  |
|  |  |  |  |  |  |  |  |
|  |  |  |  |  |  |  |  |
|  |  |  |  |  |  |  |  |
|  |  |  |  |  |  |  |  |
|  |  |  |  |  |  |  |  |
|  |  |  |  |  |  |  |  |
|  |  |  |  |  |  |  |  |
|  |  |  |  |  |  |  |  |
|  |  |  |  |  |  |  |  |
|  |  |  |  |  |  |  |  |
|  |  |  |  |  |  |  |  |
|  |  |  |  |  |  |  |  |

* Attach a separate sheet if more space is required to list the current patient medications

**12. Other comments initiated by the patient:**
